# Enhancing the purification of crocin-I from saffron through the combination of multicolumn countercurrent chromatography and green solvents

**DOI:** 10.1007/s00216-024-05228-6

**Published:** 2024-03-09

**Authors:** Mohammad Hooshyari Ardakani, Chiara Nosengo, Simona Felletti, Martina Catani, Alberto Cavazzini, Chiara De Luca, Hassan Rezadoost

**Affiliations:** 1 Department of Phytochemistry, Medicinal Plants and Drugs Research Institute, Shahid Beheshti University, G.C., Evin, Tehran, Iran; 2 Department of Chemical, Pharmaceutical and Agricultural Sciences, University of Ferrara, Via L. Borsari 46, 44121 Ferrara, Italy; 3 Department of Environmental and Prevention Sciences, University of Ferrara, Via L. Borsari 46, 44121 Ferrara, Italy; 4 Council for Agricultural Research and Economics, CREA, Via Della Navicella 2/4, 00184 Rome, Italy; 5 Center for International Scientific Studies & Collaboration (CISSC), Ministry of Science Research and Technology, Tehran, Islamic Republic of Iran

**Keywords:** Saffron, MCSGP, Preparative chromatography, Process intensification, Green chromatography, Natural compounds

## Abstract

Crocin-I, a valuable natural compound found in saffron (*Crocus sativus* L*.*), is the most abundant among the various crocin structures. Developing a cost-effective and scalable purification process to produce high-purity crocin-I is of great interest for future investigations into its biological properties and its potential applications in the treatment of neurological disorders. However purifying crocin-I through single-column preparative chromatography (batch) poses a yield-purity trade-off due to structural similarities among crocins, meaning that the choice of the collection window sacrifices either yield in benefit of higher purity or vice versa. This study demonstrates how the continuous countercurrent operating mode resolves this dilemma. Herein, a twin-column MCSGP (multicolumn countercurrent solvent gradient purification) process was employed to purify crocin-I. This study involved an environmentally friendly ethanolic extraction of saffron stigma, followed by an investigation into the stability of the crocin-I within the feed under varying storage conditions to ensure a stable feed composition during the purification. Then, the batch purification process was initially designed, optimized, and subsequently followed by the scale-up to the MCSGP process. To ensure a fair comparison, both processes were evaluated under similar conditions (e.g., similar total column volume). The results showed that, at a purity grade of 99.7%, the MCSGP technique demonstrated significant results, namely + 334% increase in recovery + 307% increase in productivity, and − 92% reduction in solvent consumption. To make the purification process even greener, the only organic solvent employed was ethanol, without the addition of any additive. In conclusion, this study presents the MCSGP as a reliable, simple, and economical technique for purifying crocin-I from saffron extract, demonstrating for the first time that it can be effectively applied as a powerful approach for process intensification in the purification of natural products from complex matrices.

**Graphical Abstract:**

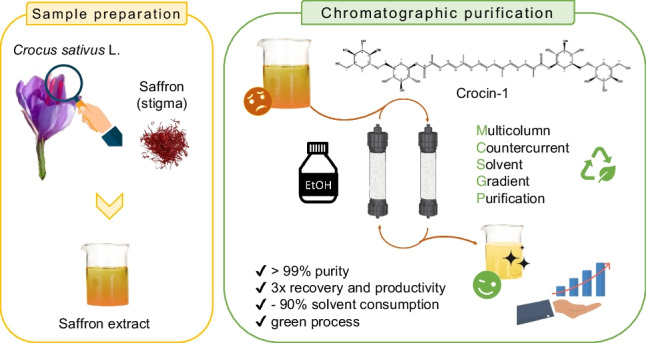

## Introduction

**Fig. 1:**
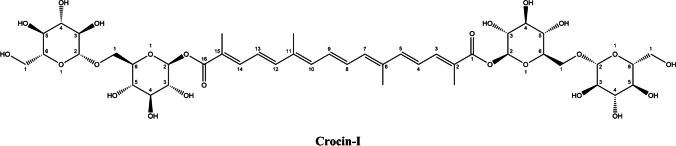
Chemical structure of crocin-I

Nature has bestowed a wide range of bioactive compounds that show potential as new drug leads. However, exploring their therapeutic value often requires development of extraction and purification techniques from complex natural sources. Saffron, the dried stigmas of *Crocus sativus* L., is a valuable source of bioactive compounds and has a long history of use as a spice and in traditional medicine [1]. The most abundant ingredients in saffron stigma are a group of carotenoid-glycosyl esters, known as crocins. The amount of total crocin can vary from 8 to 38% of the mass of dry saffron [2, 3]. However, some studies have shown that this amount can reach up to 50% [4, 5]. These differences in crocins content are mainly influenced by factors such as the country of origin, climate, nutrient and irrigation practices, drying stages, and storage conditions of saffron [6–8]. Additionally, studies have shown that crocins can be susceptible to degradation under conditions of high temperature, low pH, and exposure to light radiation [9, 10]. Therefore, it is recommended to store the samples at a low temperature and in a dark environment [11]. The number and types of sugar moieties vary among different crocin derivatives, leading to variations in their structures and potentially different biological effects. Till now, various research groups have reported a total of 47 crocins, identified through the comparison of their mass data and fragmentation behaviors [12–14]. The commonly used nomenclature for these structures is based on stereoisomers and the number of sugar moieties [15]. The most abundant type of crocin found in saffron is trans-crocetin di-(β-D-gentiobiosyl) ester (Fig. 1). It is commonly named as trans-crocin-4 due to its four glucose moieties or alternatively as crocin-I or α-crocin in some studies [16].

Crocin-I has garnered considerable attention due to its association with the quality of saffron and its potential health benefits. The pharmacological effects of crocins have been extensively studied, with numerous research highlighting their positive impact on various neurological disorders such as depression [17], Alzheimer’s disease [18], Parkinson’s disease [19], autoimmune diseases [20], cardiovascular diseases [21], bone and cartilage diseases [22], ocular disorders [23], and their potential as anti-cancer agents [24]. However, the expansion of research and preclinical and clinical studies on crocin-I is hindered by its limited commercial availability, high cost, and insufficient purity. As a result, there is a demand for scalable methods that can efficiently produce crocin-I and make it commercially accessible while ensuring high levels of purity. The successful isolation of crocin-I using two-step low-pressure chromatography [25], centrifugal partition chromatography [26, 27], high-speed counter-current chromatography [28], and macroporous resin column chromatography [29, 30] as well as combination of these techniques [31, 32] has been previously reported. Conventional chromatographic techniques for purification applications, such as for purifying crocin-I on a large scale, are mainly based on single column (= *batch*) preparative chromatography; however, they present limitations in terms of economy and efficiency, since they involve lengthy isolation times, significant use of organic solvents, and labor-intensive procedures. Additionally, the presence of structurally similar crocins can lead to poor resolution and create a dilemma whether it is better to collect narrow collection windows sacrificing yield for higher purity or to collect wider collection windows preferring a high yield rather than a high purity. This trade-off, which is a limitation associated with batch chromatography, poses a challenge in overloaded batch chromatography techniques [33–35]. An option would be to decrease the volumes of feed loaded, but this would result in very low productivity and high solvent consumption. Usually, it is preferable to give priority to purity, especially for research studies assessing pharmaceutical or biological properties of the target compound. Consequently, the purification process becomes less efficient, compromising the economic feasibility of large-scale production due to lower yields and the need for additional purification steps to achieve the desired levels of purity.

Various continuous chromatography methods have been developed since the 1960s to address these challenges [36]. The main objective of these processes is to achieve continuous loading, thereby increasing column capacity utilization and process intensification to maximize purity and yield during the purification of the compounds. In continuous chromatography, the principle is that a theoretically long column is divided into several smaller columns of identical length, which are interconnected through switching valves which can change the direction of the eluent. These smaller columns operate at steady-state conditions, reached through a high number of cycles, with optimized switching times. This technique is particularly valuable for the rapid separation of sensitive biomolecules and less stable natural compounds, such as crocins. Multicolumn countercurrent solvent gradient purification (MCSGP) technique is one of the most exciting developments of solvent gradient elution mode in continuous chromatography, which enables the separation of more than binary mixtures. This method was initially introduced in 2007 with a configuration of six identical columns [37]. Later, in optimized versions, the system was simplified using only two columns with fewer paths and valves connecting them. Today, this technique has demonstrated remarkable advancements in the purification of biological molecules such as oligonucleotides [38], peptides [33, 35], and many monoclonal antibodies and proteins [39]. However, the utilization of continuous chromatography techniques for purifying natural compounds from complex plant extracts is still rare, especially in the case of MCSGP, the benefits of this technique being still a niche topic.

To bridge this research gap, the current study aims to develop an MCSGP process for a green purification of crocin-I contained in a crude saffron extract. The greenness is even enhanced by the use of ethanol as the only organic solvent employed along all the steps, from extraction through purification.

The efficacy of this process is assessed by comparing essential parameters such as purity, recovery, productivity, and solvent consumption with those of the batch process. Additionally, to ensure a consistent feed stream during the chromatographic process, the stability of the crocin-I within the feed under different conditions was investigated. Furthermore, since there is no existing literature on the implementation of this method for purifying natural organic compounds, such as crocin-I from saffron stigma, this study represents a trailblazer in finding new sectors which could benefit from MCSGP technology.

## The MCSGP process

### Concepts of MCSGP process

**Fig. 2:**
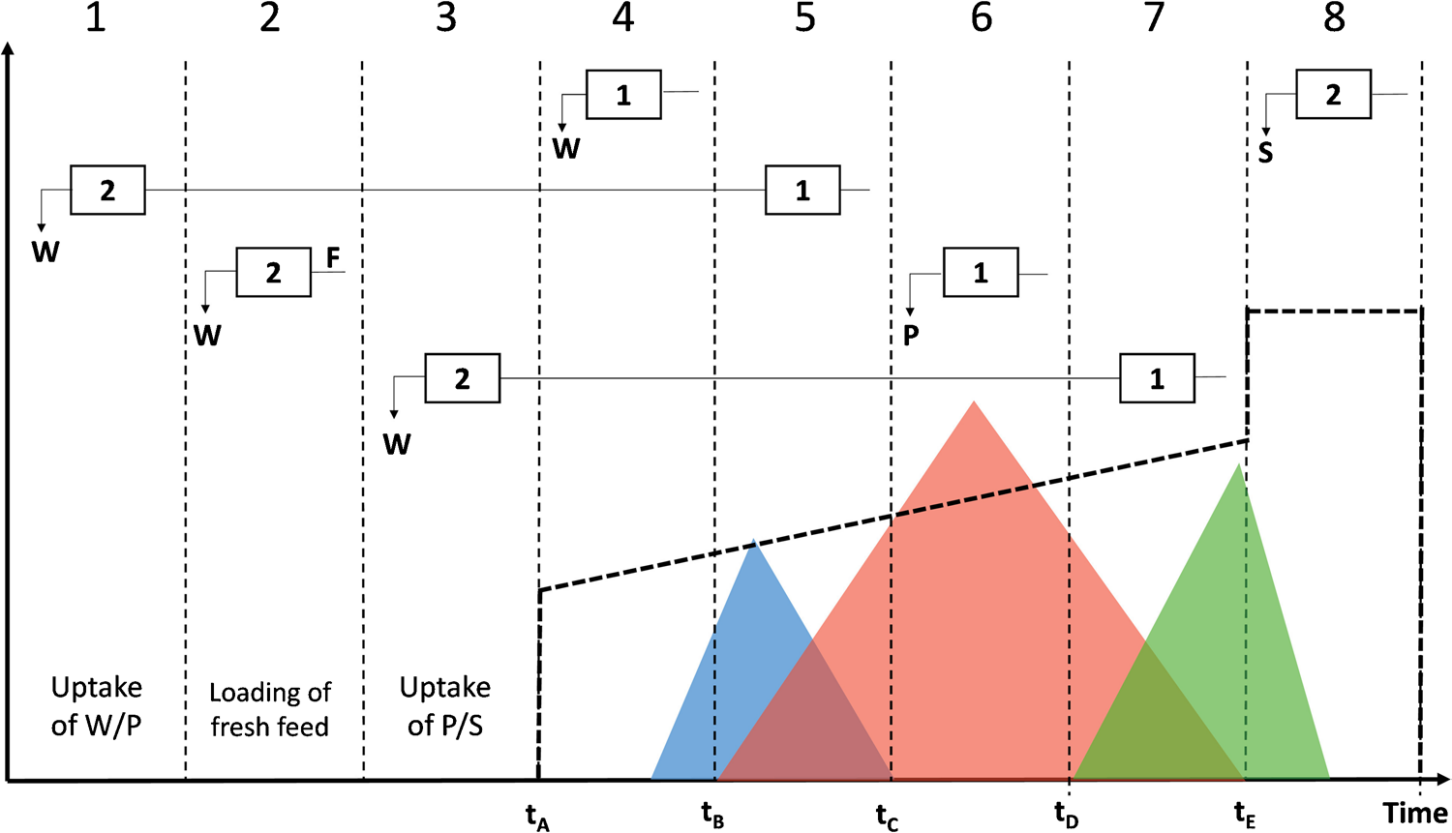
Schematic representation of batch chromatogram and its characteristic regions. The blue peak denotes weakly adsorbed impurities (W), red represents the target product peak (P), and green represents the strongly adsorbed impurities (S). Five intervals are identified where different fractions are eluting: W (t_A_ to t_B_), overlapping of W and P (t_B_ to t_C_), P (t_C_ to t_D_), overlapping of P and S (t_D_ to t_E_) and S, during the stripping. t_B_, t_C_, t_D_, and t_E_ are the characteristic switching times of the MCSGP process. t_A_ is the time where the gradient starts. Note that the loading of the target in column 2 happens in three steps: during the recycling of W/P, the loading of fresh feed (F), and the recycling of P/S. The linear gradient of the modifier is also shown. In the upper part of Fig. 2, the connection scheme between the twin columns is reported. Reproduced with permission from ref. [33]

Figure 2 provides an overview of the operational process of MCSGP. In this process, weakly (W, blue) and strongly (S, green) adsorbed impurities that co-eluted with the target compound (P, red) are both recycled using a cyclic recovery loop to achieve their separation from the target compound. This method facilitates process intensification by simultaneously maximizing purity and yield.

Briefly, one cycle of MCSGP is explained as follows. The twin-column MCSGP process utilizes a pair of identical columns that alternate between disconnected and interconnected mode to carry out a set of eight tasks. The connection scheme of the two columns is shown in the upper part (Fig. 2); the reader interested in further details is referred to literature [33]. At the beginning of gradient elution in column 1, column 2 is completing the stripping and equilibration stages (zones 4 and 8, respectively); the columns are disconnected. Successively, the valves are adjusted to connect the two columns when the overlapping region W/P begins eluting from column 1 (zone 5). This zone is contaminated with impurities but contains a large amount of target product, which is immediately uptaken by column 2 (zone 1). This automatic internal recycling process prevents waste of the target through the direct loading of overlapping zones. After the recycling of W/P, a region where only pure product is present starts eluting from column 1 and is therefore collected by the fraction collector (zone 6). In the meantime, fresh feed is injected in column 2 (zone 2); here, the columns work disconnectedly. When the overlapping region P/S begins eluting from column 1 (zone 7), the columns connect again to maintain this window into the system and load it in column 2 (zone 3). At this point, column 2 is loaded with the equivalent amount of target compound as in the batch, and the gradient can start (zone 4), while column 1 is being stripped and equilibrated. The columns have now exchanged position, indicating the completion of one switch. Once they return to their initial position, one cycle is considered complete (2 switch = 1 cycle). The times (t_A_ − t_E_) shown in Fig. 2 indicate the moments when the interconnecting valves switch position, either connecting or disconnecting the columns. Overall, the columns work as interconnected during the recycling windows, while they are disconnected during the loading of fresh feed, collection, and column regeneration. In contrast to batch processes, the feed is injected in three steps (recycling of W/P, loading of some fresh feed, recycling of P/S, represented by zones 1, 2, 3, respectively), as opposed to single step. To ensure a fair comparison between the batch and the corresponding MCSGP process, it is essential to use different column size in the two processes to achieve a similar total column volume (CV). For instance, if a column with dimensions of 250 × 4.6 mm is employed in the batch (CV = 4.2 mL), the corresponding MCSGP process may utilize twin columns with dimensions of 150 × 4.6 mm (CV_total_ = 2 × 2.5 mL = 5 mL) to maintain a comparable total column volume. These are indeed the dimensions of the columns used in this study, as explained in the “Purification process” section. Further operational details concerning MCSGP principles can be found in previous works [40–42].

### Process performance

Purity, recovery, productivity, and solvent consumption were used as the objective function to express the intensification of the purification process. All the fractions or pools collected during batch and MCSGP processes were analyzed using offline HPLC analytical protocol, described in the “Off-line analytics” section.

Peak area percentage for purity determination is unreliable when different compounds with distinct chemical structures, leading to varying absorption wavelengths in HPLC–DAD, are present in plant extracts. In saffron extract, crocins can be identified at 440 nm, while other compounds such as flavonoids and picrocrocin, which are major metabolites after crocins, are not detectable at this wavelength. For the simultaneous measurement of these compounds, a common wavelength of 250 nm was utilized. To obtain a more accurate estimation of purity, it is important to consider the presence of all compounds with absorption at different wavelengths. For fractions with unknown concentrations, using Beer-Lambert’s law can lead to the following equation to calculate the purity of crocin-I (Eq. 1):

1 $${Purity}_{C1}(\%)=\frac{{m}_{C1}}{{m}_{T}}\times 100=\frac{\frac{{A}_{C1}}{{\varepsilon }_{C1}}.{M}_{C1}}{\left[\frac{{A}_{CT}}{{\varepsilon }_{CT}}.{M}_{CT}\right]+\left[\frac{{A}_{PC}}{{\varepsilon }_{PC}}.{M}_{PC}\right]}\times 100$$

Here, *m*_C1_ represents mass of crocin-I determined by the ratio of its peak area (*A*_C1_) to its extinction coefficient (ɛ_C1_) times the molecular mass (*M*_C1_). The total mass (*m*_T_) includes the sum of two terms: the mass of the crocins at 440 nm (first term, obtained through total area of crocins (*A*_CT_) extinction coefficient of crocins (ɛ_CT_) times the average molecular mass of the four main crocins (*M*_CT_)) and the mass of other compounds at 250 nm (second term, representing picrocrocin and obtained from the total area of picrocrocin and flavonoids (*A*_PC_), extinction coefficient of picrocrocin (*ɛ*_PC_), and molecular mass of picrocrocin (*M*_pc_)). The extinction coefficient for both crocin-I and crocins was assumed 89,000 M^−1^ cm^−1^ at 440 nm and for picrocrocin was assumed to be 10,100 M^−1^ cm^−1^ at 250 nm, as reported in literature [43]. The average of molecular mass of the four main crocins was 918.93 as reported in previous study [44]. It’s important to note that this method is an approximate but efficient approach utilizing HPLC analysis.

The process recovery, also known as yield, is the ratio between the mass of the target compound (crocin-I, CI) that is successfully recovered within the specified purity requirements and its mass initially loaded in the feed:

2 $${Recovery}_{CI}(\%)=\frac{{m}_{recoverd}}{{m}_{loaded}}\times 100$$

Productivity refers to the rate at which the target compound is purified within the specified purity, taking into account the duration of the method (*T*_run_) and the total volume of the stationary phase (CV), defined as the geometrical volume of the cylindrical column:

3 $${Productivity}_{CI}\left(\frac{g}{L\cdot h}\right)=\frac{{m}_{recoverd}}{{T}_{run}\cdot CV}$$

Finally, solvent consumption refers to the amount of solvent (V_s_) used by the purification method, including the total organic and aqueous mobile phases necessary to complete all the steps of the process, to purify the recovered mass of the target product while maintaining the desired purity:

4 $$Solvent\;consumption \left(\frac{L}{g}\right)=\frac{{V}_{s}}{{m}_{recoverd}}$$

It must be highlighted that these equations hold valid for both the batch and MCSGP processes. The difference is that, for the MCSGP, these parameters can be calculated based on the cycle. In that case, the mass recovered corresponds to the sum of the mass collected from zone 6 of Fig. 2 for the two switches. Additionally, when determining productivity, the column volume (CV) to be used is twice the CV of one of the two identical columns.

## Materials and methods

### Chemicals and solvents

Crocins with purity 97.20% were isolated in our previous study [44] and were used as working standard in this study. HPLC-grade solvents, including acetonitrile (ACN) and ethanol (EtOH), were purchased from Merck (Milan, Italy). Ultrahigh pure water was purified by a Millipore Milli-Q system.

### Saffron extraction

Ten grams of pulverized dried saffron stigma from Iran was mixed with 1 L of an 80% ethanol and water composition, as a green extraction solvent. The mixture was then sonicated for 10 min at room temperature in an ultrasonic bath. After decanting the separated mixture, the wet powder underwent an additional extraction with 1 L of fresh 80% ethanol/water solution to maximize the extraction efficiency. Subsequently, the resulting supernatant was concentrated using a vacuum rotary evaporator and then dried with a freeze dryer. Finally, 5.9 g of the resulting extract powder were obtained and were stored at + 4.0 °C.

### Feed stability study

In order to investigate the stability of crocin-I, a stock solution was prepared by dissolving 100 mg of crude saffron extract in 20 ml of mixture of 95% water and 5% ethanol. The resulting extract was then divided equally between two vials, both of which were covered with aluminum foil. One vial was stored at room temperature, while the other was placed in a refrigerator set at a temperature range of 6–8 °C. Sampling was conducted at regular intervals from both vials, and after dilution, a concentration of 0.1 g/L was used to analyze the rate of degradation using offline HPLC analytical protocol, described in the “Off-line analytics” section.

### Purification process

The feed is prepared by dissolving the crude extract in a solution through agitation for 30 min, with a composition of 5% EtOH and 95% water, to reach a final concentration of crocin-I of 1.3 g/L. The concentration was determined using the offline HPLC analytical protocol described in the “Off-line analytics” section.

**Fig. 3:**
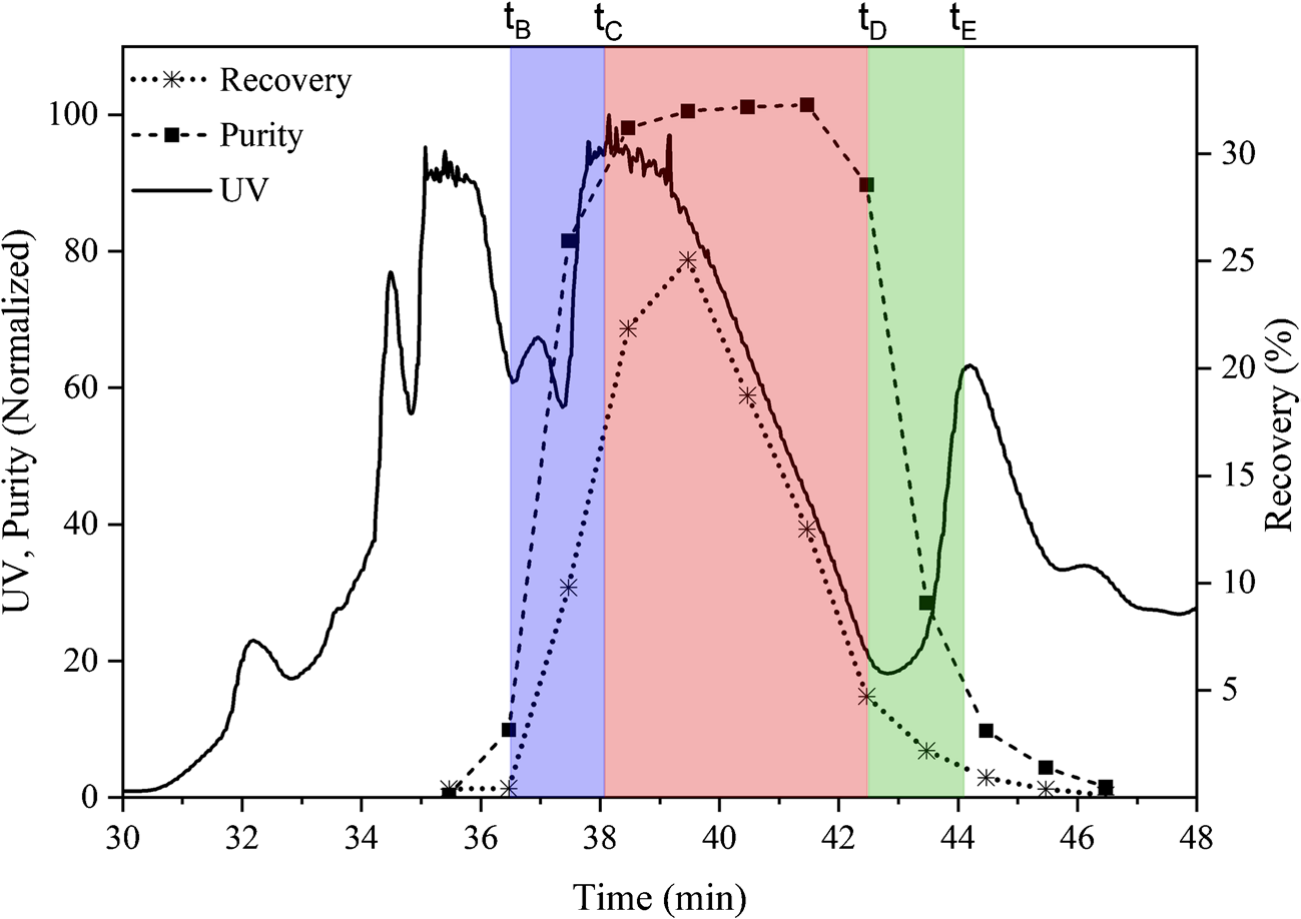
Design batch chromatogram (at 350 nm), reporting purity and recovery of crocin-I. Also recycling intervals of W/P (blue) and P/S (green) and target product collection (red) chosen for the MCSGP process design and the relative switching times (at the top of the graph) are highlighted

The purification of crocin-I was conducted on a Contichrom CUBE system (Chromacon YMC, Zurich, Switzerland) equipped with 2 dual-head pumps, 4 multi-position valves, and two external detectors set at 350 nm (to prevent detector saturation at the *λ*_max_ of crocins (440 nm) and serve as an intermediary wavelength between the target compound and impurities). Fraction collection was performed using a Foxy R1 fraction collector (KNAUER, Berlin, Germany). Both the batch and MCSGP processes employed the same stationary phase, namely Daisogel-SP-120–10-C18-Bio columns with a particle size of 10 μm. The dimensions of the batch column used in this study were 250 × 4.6 mm, while the MCSGP process was carried out using two smaller columns with dimensions of 150 × 4.6 mm. The purpose of using these smaller columns was to ensure that the column volume remained similar when comparing the two processes (4.2 mL of CV for the batch purification and 2 × 2.5 = 5 mL of CV for the MCSGP process). For the linear gradient, two mobile phases consisting of a mixture of water and ethanol were used. Mobile phase A (MPA) contained 95% water and 5% ethanol, and mobile phase B (MPB) contained 95% ethanol and 5% water. In both the batch and MCSGP processes, the columns were initially equilibrated with 3 column volumes (CV) of MPA at a flow rate of 1 ml/min. Following equilibration, the feed solution (a saffron extract with a concentration of 5 g/L and containing 1.3 g/L crocin-I) was loaded at a flow rate of 1.5 ml/min. In the batch and MCSGP (for the first switch), the feed volume was set to 7.5 CV (so that the mass injected corresponds to 1% of the stationary phase volume, e.g., 10 mg product/1000 μL SP). In subsequent switches in MCSGP, the loading rate was adjusted to compensate for the crocin-I leaving the system, ensuring a consistent feed stream for both columns. Figure 3 represents the design batch chromatogram necessary to set up the MCSGP windows, where the overlapping regions are colored in blue and green and the collection pool is reported in red. Within each window, a certain amount of product elutes, and these amounts are calculated through the offline analysis. For instance, at each MCSGP switch, some product leaves the system either in W or in P or in S; this amount must be reinjected in order to keep the loading of the system constant along the process. To calculate the re-injected volume (V_re_), Eq. 5 was used:

5 $${V}_{re}=\left(\frac{{M}_{w}+{M}_{S}+{M}_{p}}{100}\right)\times {V}_{batch}$$

where *M*_W_ represents the percentage of the crocin-I wasted in the regions *t* < *t*_B_, *M*_S_ represents the percentage of the crocin-I that wasted in the regions *t* > *t*_E_, and *M*_P_ the percentage collected in the target product window depicted by the red region in Fig. 3 (*t*_C_ < *t* < *t*_D_,). *V*_batch_ represents the batch loading in single column. *t*_A_ (reported only in Fig. 2) is instead the time when the gradient starts.

In this study, a batch loading was performed by loading 7.5 CV as explained earlier. Overall, a total of 78.8% of the mass leaves the system, with 2.7% exiting through the waste streams (W + S) and 76.1% is collected as pure product (P). Therefore, to ensure a constant feed loading, a volume equivalent to 5.9 CV (meaning 78.8% of 7.5 CV) was loaded in each switch after the first one. The remaining 21.2% was loaded during the recycling steps. After the feed has been loaded, a linear gradient from 22 to 44% mobile phase B was applied at a flow rate of 0.6 ml/min for 7 CV. In the batch operation, fractions were collected every minute, while in the MCSGP process, 2 fractions were collected per cycle (one fraction per switch corresponding to the red area (P) shown in Fig. 3). Finally, the columns were stripped with 100% MPB at a flow rate of 1 ml/min for 3 CV, effectively removing strongly absorbed metabolites from the stationary phase.

In summary, the MCSGP process utilized three primary stages. Initially, during the start-up stage, the feed for the first switch was adjusted to 7.5 CV, equivalent to the quantity used in the batch run. Subsequently, in the main cycles (five cycles in this study), each column received a feed of 5.9 CV. Finally, known as the shut-down stage, no additional feed was supplied, and the columns were completely eluted, marking the completion of the process.

### Off-line analysis

The saffron crude extract, each fraction, and the purified crocin-I were analyzed using an AZURA HPLC system (KNAUER, Berlin, Germany) equipped with a PDA detector, binary HPG pump, autosampler, and column thermostat. A Titan® C18 column (100 × 3 mm, 1.9 μm) was utilized. The mobile phases consisted of water (A) and acetonitrile (B), both containing 0.05% TFA and flow rate of 0.3 mL/min. The gradient program started at 10% B, increased to 50% B over 15 min, rapidly reached 100% B in 2 min, and remained constant for 3 min to clean the column. The initial conditions (10% B) were restored within 2 min, allowing the column to re-equilibrate for 5 min. The detection wavelengths were set at 440 nm and 250 nm, with an injection volume of 2 µL. Quantitative analysis was performed using an external standard calibration curve.

## Results and discussion

### Feed stability and composition

**Fig. 4:**
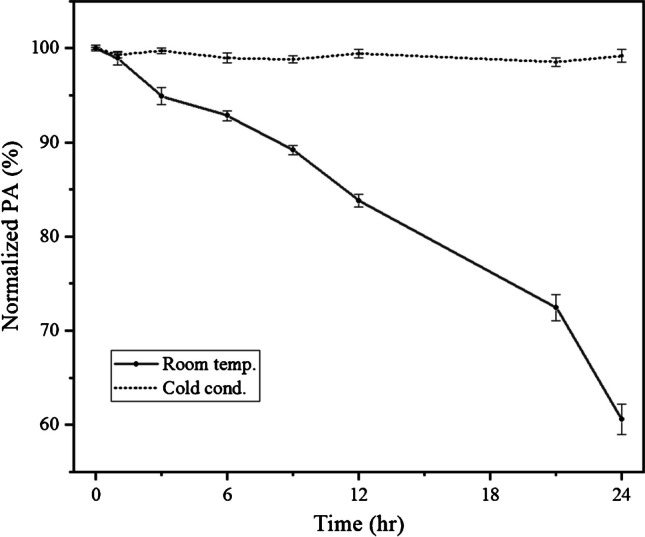
Degradation kinetic of the crocin at different temperatures, expressed as the variation of the normalized peak area within time

Ensuring consistency in the quality of the input feed is crucial for obtaining reproducible results in the output of the MCSGP process. Therefore, maintaining a constant quality extract is of utmost importance. To investigate the stability of crocin-I, the decrease in its peak area at 440 nm was measured over a 24-h period at two different temperatures: room temperature and cold condition (6–8 °C). To ensure the reliability of the findings, the experiment was repeated on two consecutive days. The graph presented in Fig. 4 demonstrates that the feed stored under cold conditions remained stable for at least 24 h. In contrast, the feed stored at room temperature exhibited a 40% reduction in its crocin-I content after 24 h. Therefore, by storing the feed at a temperature of 6 to 8 °C during the process and renewing the feed at least once every 24 h, it is possible to ensure a stable feed throughout the purification process.

**Fig. 5:**
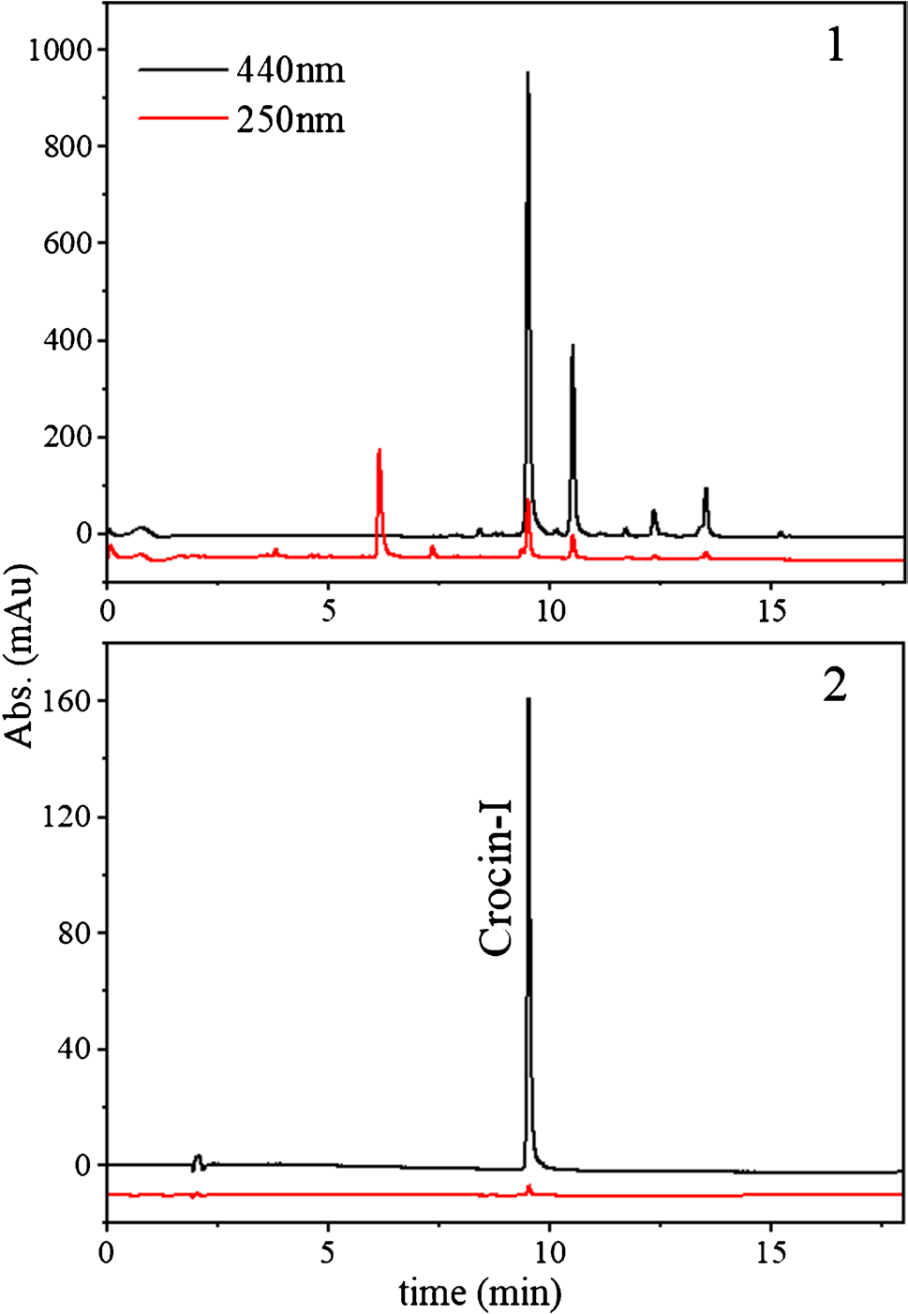
Analytical HPLC chromatograms of (1) crude saffron extract and (2) purified crocin-I at two wavelengths (440 and 250 nm)

Figure 5 illustrates the RP-HPLC chromatogram displaying the saffron metabolite profile (the crude feed before purification) at two specific wavelengths. At 440 nm, which corresponds to the absorption region of crocin-I and other crocins, it is possible to identify compounds eluting after the target product which are the strongly adsorbed impurities referred to as “S.” On the other hand, the compounds eluting before the target product are weakly adsorbed impurities named “W.” Part of the impurities labeled as “W” consists of crocins with smaller retention times observed at 440 nm, while most of the impurities are associated with picrocrocin, flavonoids, and other compounds in the absorption region of 250 nm. The initial purity of crocin-I in the feed, estimated using the method described in the “Performance of process” section, was found to be 27%.

### Design batch process

**Fig. 6:**
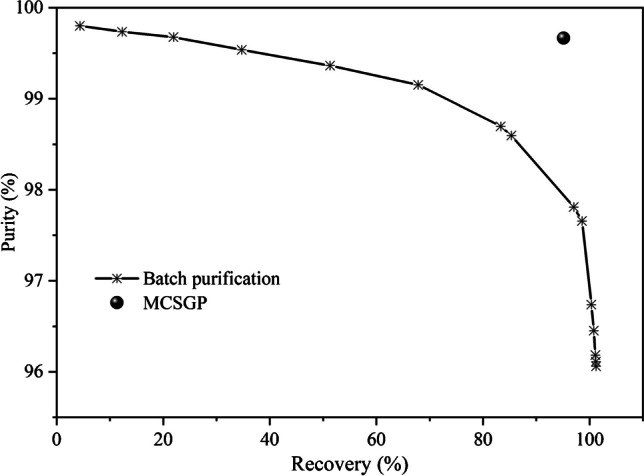
Pareto curve obtained in batch purification with a 25-cm column and the point representing the MCSGP performance at steady state

To demonstrate the process intensification and evaluate parameters such as purity, recovery, productivity, and solvent consumption, a traditional batch purification of the saffron crude extract was conducted using a 250 × 4.6 mm C18 column as described in the “Purification process” section. The primary objective of this investigation was to examine the relationship between the purity of crocin-I and its recovery under various gradient conditions. The curve that put in relation purity and recovery is the so-called Pareto curve, which shows how the purity decreases when enlarging the collection window, meaning while increasing the recovery. The point in the upper left part of the graph (Fig. 6) corresponds to the purest fraction, which also has a low recovery, while the point at the right bottom represents the purity obtained when the whole peak is collected. By comparing the Pareto curves of different gradient elution methods, the optimal gradient elution was determined. A shallower slope of the Pareto curve indicated that the purity decreased at a slower rate with increasing recovery. Among the methods tested, the condition and gradient described in the “Saffron extraction” section were selected as the optimal elution condition. This resulted in the Pareto curve shown in Fig. 6, which indicates the purity values of the collected target product and the corresponding fraction recoveries during batch purification.

Furthermore, the same methodology was applied on a short column (15 cm), which was chosen as the design batch chromatogram and served as the foundation for developing the MCSGP process. The flow rate, gradient slope, and composition of mobile phases for the short column were kept consistent, while the volume of the feed and mobile phases in each step was adjusted according to the volume of the short column. Figure 3 presents the corresponding chromatogram, along with the purity and recovery profiles of crocin-I in the various fractions.

Therefore, while the batch run on the short column is used to decide the recycling and collection windows, the batch run on the long column is taken as a reference to make a comparison with the MCSGP performance.

### The MCSGP process

The successful design of an MCSGP process requires precise selection of switching times and stable feed loading. Based on the design batch chromatogram run on the 15-cm column (Fig. 3), and its Pareto curve (Fig. 6), various combinations of switching times from *t*_B_ to *t*_E_ were tested. For this study, the following time intervals were selected: *t*_A_ = 26.5 min, *t*_B_ = 36.5 min, *t*_C_ = 38.1 min, *t*_D_ = 42.5 min, and *t*_E_ = 44.1 min. These times were highlighted on the design batch chromatogram in Fig. 3 to indicate the recycling and collection windows associated with each time.

**Fig. 7:**
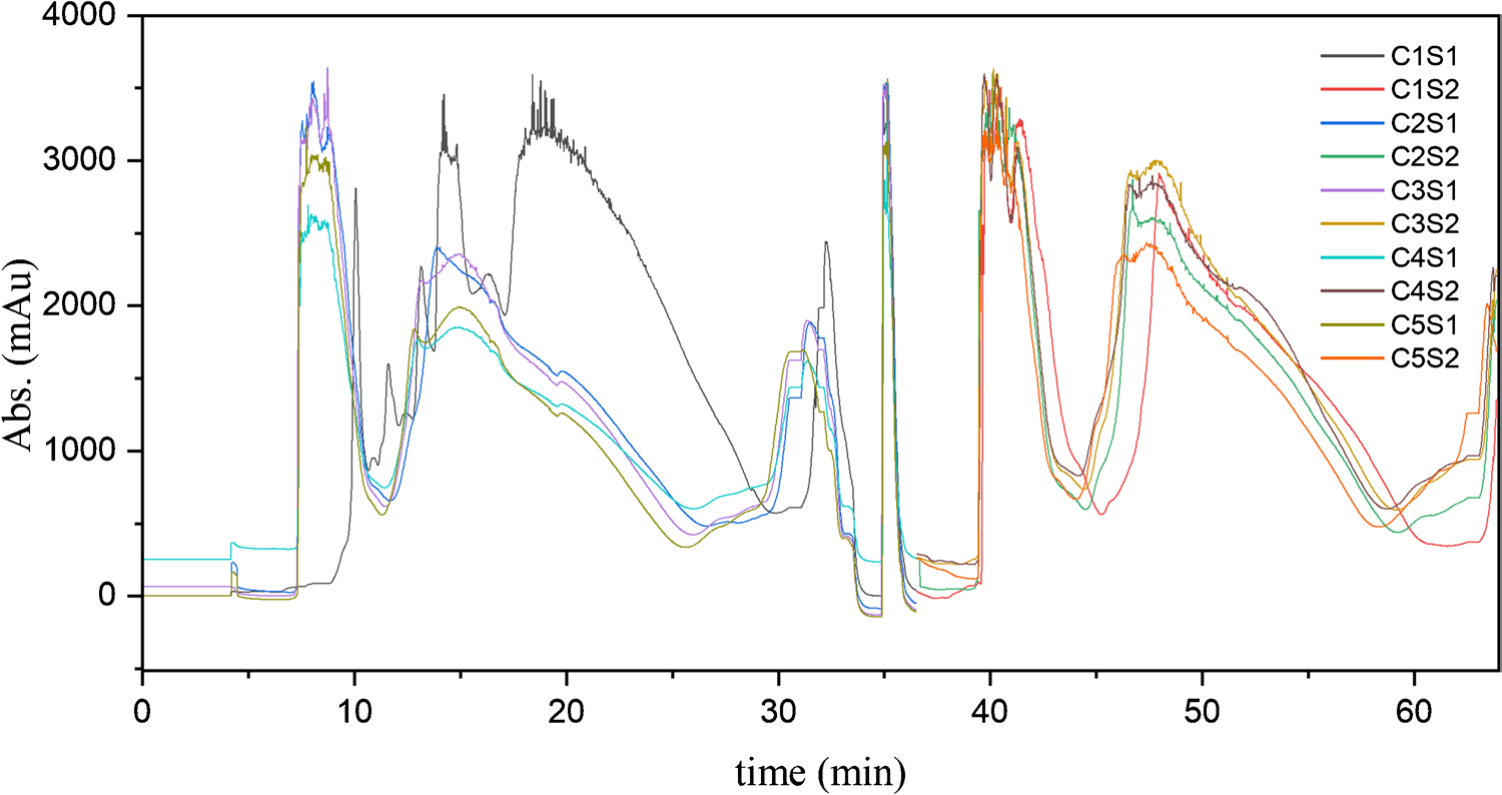
Overlaid of the UV signals (at 350 nm) measured at the outlet of the two columns of the MCSGP unit for 5 cycles (10 switches). C=number of cycle, S=number of switch

In this study, the MCSGP was employed for five cycles, resulting in a total of 10 switches. Except for the first switch (shown in black in Fig. 7), the UV signals recorded at the outlet of both columns were almost overlaid.

The fact that the signal pattern observed during the first cycle differed from the subsequent cycles is related to the different composition of the injected feed into the columns during the initial switch. This initial switch represented the batch separation condition, where the column was loaded without recycling the W/P and P/S overlapping zones in the input feed. However, starting from the second switch, the cycles exhibited a consistent and nearly identical pattern, confirming the establishment of a steady-state condition, meaning that the system performance does not change with time. This finding is further supported by the calculated average purity and recovery values, along with the standard deviation, across the five cycles, as presented in Table 1. These average parameters represent a single point on the Pareto curve graph, corresponding to the performance of the MCSGP process once steady state is reached, given the switching times chosen. The comparison between the performance obtained in batch (25-cm column) and in MCSGP is displayed in Fig. 6.

**Table 1:** Comparison of the performance parameters of the batch and MCSGP processes and summary of process intensification by MCSGP

	Total CV (mL)	Purity (%)	Recovery (%)	Productivity (g/L/h)	Solvent Cons. (L/g)
Batch run	4.2	99.7 ± 0.0	21.9 ± 0.2	1.4 ± 0.0	46.4 ± 0.0
MCSGP run	5	99.7 ± 0.1	95.1 ± 1.0	5.7 ± 0.5	3.5 ± 0.0
Process intensification			+ 334%	+ 307%	− 92%

Table 1 provides a numerical comparison of the performance parameters achieved by the two purification methods for crocin-I.

When comparing the two approaches at the same level of purity, the batch purification method yielded only a 21.9% recovery of a hypothetical pool of crocin-I, whereas the MCSGP demonstrated a significantly higher recovery rate, equal to 95.1%. This implies that the MCSGP process led to a 334% increase in the recovery of the target product. This improvement is clearly visible in Fig. 6, where the point related to the performance of MCSGP lies in the upper right part of the graphic and above the batch Pareto curve, meaning that at the same purity a higher yield is obtained. One of the key factors contributing to this enhanced recovery is the ability of the MCSGP technique to recycle the overlapping portions by reintroducing them into the system for further separation. In addition to recovery, also process productivity and solvent consumption are vital factors to consider, since they represent a measure of the economic feasibility and environmental impact of the process, respectively.

Productivity measures the quantity of purified crocin-I produced per unit time and unit column volume. The findings revealed a significant intensification in productivity, with the batch method yielding 1.4 g/L/h, whereas the MCSGP achieved a substantially higher productivity of 5.7 g/L/h. This corresponds to a 307% boost in productivity for the MCSGP. Coming to the solvent consumption, in the case of the batch process, again considering a hypothetical pool of crocin-I with same purity as the MCSGP process, the amount of solvent used per gram of purified crocin-I is 46.4 L/g. However, in the steady-state operation of MCSGP, this value is significantly reduced to 3.5 L/g, thanks to the utilization of two 15 cm columns in the MCSGP unit instead of a single 25-cm batch column, resulting in an impressive 92% reduction in solvent consumption.

## Conclusion

In this study, a scalable and green process for the extraction and purification of crocin-I from saffron stigma were successfully achieved. The feed for purification was an ethanolic extract obtained through the ultrasonic method. To ensure a consistent feed stream throughout the procedure, the degradation rate of target compound was investigated. It was observed that the feed remained stable for at least 24 h under cold conditions. The purification process, through a single-column (batch) preparative chromatography approach, encountered challenges in achieving high recovery and purity simultaneously. However, the MCSGP addressed this issue by internally recycling the overlapping zones of the target compound and co-eluted impurities. This led to a significant improvement in the recovery, increasing from 21.9% in the batch to over 95% in the MCSGP process while maintaining the same purity grade (99.7%). Moreover, the MCSGP demonstrated a significant enhancement in productivity, achieving a rate of 5.7 g/L/h compared to 1.4 g/L/h for the batch purification process, resulting in a remarkable 307% process intensification. In addition, the amount of solvent used in batch to MCSGP processes reduced by 92%, from 46.4 to 3.5 L/g.

In conclusion, the successful implementation of the MCSGP process does not only help to overcome challenges in purifying complex ternary mixtures by boosting the process performance, as already proven for biopharmaceuticals, but also opens possibilities for purifying natural compounds from complex matrices. The greenness of the MCSGP technique, related to the intensification of process performance, namely recovery, productivity, and solvent consumption, is even more enhanced by the use of ethanol, an organic solvent traditionally considered greener than, for instance, acetonitrile, which is by far the most employed solvent in reversed-phase liquid chromatography [45, 46]. The combination of these two aspects could represent a milestone in the low-environmental-impact, scalable, and economically viable production of valuable natural compounds. Indeed crocin-I, as reported in this study, was the trailblazer molecule to prove for the first time the effectiveness of this continuous process also for natural matrices, but similar outcomes are expected also for different plant (and food) compounds.
